# Pyriproxyfen Exposure Impairs Cognitive Parameters and Alters Cortisol Levels in Zebrafish

**DOI:** 10.3389/fnbeh.2020.00103

**Published:** 2020-06-17

**Authors:** Darlan Gusso, Gustavo Kellermann Reolon, Jonas Brum Gonzalez, Stefani Altenhofen, Luiza Wilges Kist, Mauricio Reis Bogo, Carla Denise Bonan

**Affiliations:** ^1^Programa de Pos-Graduacao em Biologia Celular e Molecular, Escola de Ciencias da Saude e da Vida, Pontificia Universidade Catolica do Rio Grande do Sul, Porto Alegre, Brazil; ^2^Programa de Pos-Graduacao em Medicina e Ciencias da Saude, Escola de Medicina, Pontificia Universidade Catolica do Rio Grande do Sul, Porto Alegre, Brazil

**Keywords:** locomotion, memory, cortisol, pyriproxyfen, zebrafish

## Abstract

Pyriproxyfen is one of the most used larvicides and insecticides; it acts as an analog of juvenile insect hormone (a growth regulator). It is highly toxic during all stages of mosquito development, suppresses metamorphosis, and interferes in insect reproduction and proliferation. Pyriproxyfen and its main metabolite have been shown to affect brain development in rodents. This compound is employed mainly to eliminate outbreaks of the genus *Aedes*, even in potable water. Despite the increasing number of toxicological studies about larvicides and insecticides—with an indication of continuous use—there have been few studies about the effects of pyriproxyfen in non-target species such as fish. This study evaluated the effects of pyriproxyfen on behavioral, cognitive, and endocrine parameters in zebrafish. We exposed adult zebrafish to different pyriproxyfen (Pestanal^®^) concentrations (0.125, 0.675, and 1.75 mg/l) for 96 h. We analyzed behavioral parameters, memory, cortisol levels, and gene expression of glucocorticoid receptor (*gr*) and corticotrophin-releasing factor (*crf*) after pyriproxyfen exposure. This exposure did not alter locomotion (distance or mean speed), anxiety-like behavior (latency to enter to the top zone of the tank or time in the top zone of the tank), and social or aggressive behavior. However, there was impaired inhibitory avoidance memory at all tested pyriproxyfen concentrations. Cortisol levels were reduced in exposed groups when compared to control or vehicle. However, *gr* and *crf* gene expression in pyriproxyfen-treated animals were unaltered when compared to control or vehicle groups. Taken together, these findings indicate that pyriproxyfen may induce cognitive impairment and altered cortisol levels in zebrafish, a non-target species.

## Introduction

Pyriproxyfen is a larvicide used mainly for the control of mosquitoes of the genus *Aedes*. Despite its broad use, pyriproxyfen has been shown to affect the central nervous system. The main pyriproxyfen metabolite, 4’OH-pyriproxyfen, alters gene expression in mouse neurospheres cultures, indicating possible consequences on neurogenesis and brain morphology (Spirhanzlova et al., 2018). Moreover, rat pups chronically exposed to pyriproxyfen showed arhinencephaly and reduced brain weight (Evans et al., 2016). Pyriproxyfen has been approved by the World Health Organization as safe for use even in potable water [World Health Organization (WHO), 2006]. It is highly toxic during all stages of mosquito development, suppresses metamorphosis (Chłopecka et al., 2018), and interferes in insect reproduction and proliferation. It acts as an analog of juvenile insect hormone, a growth regulator [Ishaaya and Horowitz, 1992; World Health Organization (WHO), 2006; Chłopecka et al., 2018]. Pyriproxyfen may also act as an acetylcholinesterase inhibitor in fish (Araújo et al., 2018; Maharajan et al., 2018).

The use of larvicides and insecticides in freshwater can affect non-target species (Ghelichpour et al., 2018), such as fish, and trigger an imbalance in the endocrine system and, consequently, alter the release of cortisol (Koakoski et al., 2014). Cortisol is a stress biomarker and is a primary product of the hypothalamic-pituitary–interrenal (HPI) axis in fish and of hypothalamic–pituitary-adrenal (HPA) axis in mammals (Pijanowski et al., 2015; Adam et al., 2017). A stressful signal induces the release of corticotrophin-releasing factor (CRF) in the neurosecretory preoptic area (NPO) in fish, which is homologous to the paraventricular nucleus of the hypothalamus (PVH) in mammals (Herget et al., 2014). In response to CRF, the pituitary releases adrenocorticotropic hormone (ACTH) into the bloodstream; this hormone, in turn, stimulates the release of cortisol from interrenal cells located within the kidney head of fish. Cortisol is secreted and binds to the glucocorticoid receptor (GR; Bury and Sturm, 2007; Baker et al., 2015). The stress response between humans and zebrafish has a high level of similarity because both produce cortisol as a hormone in response to stress (Barcellos et al., 2007; Alsop and Vijayan, 2008; Katsu et al., 2018). Insecticides can cause changes that modify the endocrine response of fish (Zhang et al., 2015). These modifications may cause deregulation on the HPI axis and promote important behavioral alterations for fish (Boscolo et al., 2018), such as changes in memory consolidation (Manuel et al., 2014; Bennion et al., 2015). Memory consolidation prepares animals to defend themselves from dangerous situations, such as predation risk (Oliveira et al., 2017), *via* an aversive memory (Gerlai, 2016). In the memory process, several distinct mechanisms can be affected by different chemical compounds (Altenhofen et al., 2017a; Woodcock et al., 2018) and traumatic effects (Manuel et al., 2014). Studies in mammals (rats and humans) have demonstrated that negative emotions are regulated by the hippocampus (Goosens, 2012). Fish have a high capacity to retain aversive and spatial memory similar to the functions of the hippocampus in mammals (Gerlai, 2016). Indeed, fish can preserve important emotional information that leads them to avoid predation, objects, places, and disputes (Oliveira et al., 2013; Gaspary et al., 2018).

The zebrafish (*Danio rerio*) is a highly reliable model. It usually takes zebrafish less than 2 h to learn a task (Aoki et al., 2015), and the retention time can exceed 24 h (Altenhofen et al., 2017b; Bridi et al., 2017). Despite the increasing number of toxicological studies about larvicides and insecticides with an indication for continuous use, there are few studies about the effects of pyriproxyfen in fish, which is an important model for pollution studies (Charreton et al., 2015; Kais et al., 2015). Thus, we performed experiments with zebrafish, a teleost that shares a high degree of gene sequence and functional homology with mammals (around 70%), including humans. This fish has a complex behavioral repertoire and many zootechnical advantages, such as easy maintenance and high fertility (Howe et al., 2013; Orger and de Polavieja, 2017). Further, it is a well-established model for studying behavior (Egan et al., 2009; Zhang et al., 2015; Gerlai, 2017), memory (Al-Imari and Gerlai, 2008; Grossman et al., 2011; Gerlai, 2016; Bridi et al., 2017; Gaspary et al., 2018), developmental biology (Nery et al., 2014), cognition (Grossman et al., 2011; Zimmermann et al., 2015), endocrinology (Abreu et al., 2015; Idalencio et al., 2015), and toxicology (Altenhofen et al., 2017a). Given that little is known about the action of pyriproxyfen on non-target organisms, its evaluation in these animals becomes essential to understand this insecticide’s mechanism(s) of action. Therefore, this study aimed to evaluate the effects of pyriproxyfen on behavioral, cognitive, and endocrine parameters in adult zebrafish.

## Materials and Methods

### Animals and Housing

We used 461 animals from our breeding colony in equal proportions (male/female) of adult (6–7 months) wild-type zebrafish. Fish were kept in automated recirculating systems (Zebtec, Tecniplast, Italy), which contain reverse osmosis filtered water, at the recommended temperature (28°C ± 2°C), pH (7.0–7.5), conductivity (300–700 μS), hardness (80–300 mg/l), ammonia, nitrite, nitrate, and chloride levels for the species (Westerfield, 2000, 2007). We maintained animals on a light/dark cycle of 14/10 h and fed them with commercial flakes (TetraMin Tropical Flake Fish^®^) three times a day. Fourteen days post-fertilization, we supplemented the diet with brine shrimp (Westerfield, 2000). All protocols were approved by the Institutional Animal Care Committee from Pontificia Universidade Catolica do Rio Grande do Sul (CEUA-PUCRS, protocol number 7546/2016). We registered this study in the Sistema Nacional de Gestão do Patrimonio Genetico e Conhecimento Tradicional Associado—SISGEN (Protocol No. A3B073D).

### Pyriproxyfen Exposure

For all experiments, we exposed animals to one of the following conditions: water (control group, *n* = 94), 0.5% dimethyl sulfoxide (DMSO, *n* = 95; CAS number 67-68-5, purity > 99%; vehicle; (Hallare et al., 2006; Nery et al., 2014), or 0.125 (*n* = 94), 0.675 (*n* = 90), or 1.75 mg/l (*n* = 88) pyriproxyfen (PESTANAL^®^, CAS number 95737-68-1 purity 99.3%; Sigma–Aldrich, St. Louis, MO, USA). Pyriproxyfen has low water solubility, and the concentration of 0.5% DMSO was chosen as a diluent according to previous studies (Padilla et al., 2012; Truong et al., 2016). The exposure time for all groups was 96 h. We chose these pyriproxyfen concentrations based on recommended use concentrations from the World Health Organization and Brazilian Ministry of Health (recommended concentration for drinking water: 0.01 mg/l; World Health Organization (WHO), 2006; Brasil, Ministério da Saúde, 2014) and previous studies in zebrafish (0.16, 0.33 and 1.66 mg/l; Maharajan et al., 2018). Immediately after exposure, we subjected all groups to behavioral tests and collected tissues for molecular analysis. We used each animal for just one experiment. We obtained the water used for the experiments from enriched reverse osmosis, as indicated for zebrafish (Westerfield, 2007). After each test session, we completely changed the water. During the exposure period, there were no differences in the survival of the exposed groups.

### Liquid Chromatography Coupled to Tandem Mass Spectrometry (LC-MS/MS) for Pyriproxyfen Analysis

To evaluate the effective pyriproxyfen concentration in the exposure water at the end of treatment, immediately after the end of the exposure period, we collected a water sample from each aquarium and placed it in a 2 ml polypropylene tube. We froze samples and stored them at −80°C. For analytic quantification, we thawed samples at room temperature and vortexed them for 30 s. We transferred a 1.0 ml aliquot of each sample to 1.5 ml centrifuge tubes and added 0.1 ml of methanol to dissolve any insoluble pyriproxyfen particles and aid precipitation of the suspended materials. After further agitation, we centrifuged the tubes at 14,000 rpm at 4°C for 20 min, transferred the supernatant to 2 ml glass vials, and analyzed them with LC-MS/MS.

The LC-MS/MS system comprised an Aquity I-Class UPLC (Waters Corp.) coupled to a Xevo TQ-S micro MS/MS (Waters Corp.). We performed the separation in the reverse phase with a C18 Zorbax Bonus-RP Rapid Resolution chromatographic column (2.1 × 50 mm, 1.8 μm; Agilent Technology), preceded by a guard column of the same material. The mobile phase consisted of (A) 0.1% formic acid and (B) 0.1% formic acid in acetonitrile, 0.4 ml/min flow at 50°C. The gradient consisted of 15% B up to 1.2 min, increasing to 85% at 3.1 min, and remaining at that rate for 1 min. Then, we resumed the initial proportion and performed stabilization for 1 min. We next injected samples (5 μl) into the system with the use of an autosampler.

We performed MS in positive mode with an electrospray source operated at 550°C desolvation temperature, 1,500 V capillary, 50 V cone, and 20 V collision energy. We adjusted the spectrometer to monitor the m/z 322–185 and m/z 322–96 transitions, the first quantization transition, and the last qualification transition in multiple reaction monitoring (MRM) mode. The retention time of the pyriproxyfen was 3 min; we quantified its concentration using the regression equation of the built-in calibration curve with a broad concentration range. The employed curve model was quadratic, with a coefficient of determination (R^2^) greater than 0.99. We adapted the analysis from a previous work (Liu et al., 2019).

### Novel Tank Test

We placed animals individually in experimental tanks (30 cm long × 15 cm high × 10 cm wide) with water and recorded them for 6 min. After 60 s of habituation, we analyzed the locomotion and exploratory patterns of the fish using EthoVision XT^®^ tracking software (version 11.5, Noldus, Wageningen, The Netherlands) at a rate of 30 positions per second (Altenhofen et al., 2017b). We evaluated the following behavioral parameters: distance (m), mean speed (m/s), time spent in the upper zone (bottom vs. upper levels; s), crossings, and latency to the upper zone (s). We considered the time spent in the upper zone and latency to reach the upper zone as indicators of anxiety-like behavior (Levin et al., 2007).

### Aggressive Behavior

We explored aggressive behavior following the method described by Gerlai et al. (2000) and adapted by Bridi et al. (2017). The experimental tank dimensions were: 30 cm long × 15 cm high × 10 cm wide. We placed a mirror (45 cm × 38 cm) at the side of the tank at an angle of 22.5° to the tank’s back wall, so that the left vertical edge of the mirror touched the side of the tank and the right edge was farther away. Thus, when the experimental fish swam to the left side of the tank, their mirror image appeared nearest to it. We added an individual zebrafish to the tank and allowed it to acclimate for 60 s; we subsequently recorded aggressive behaviors conducted toward its mirrored image for 5 min. We evaluated behavior using EthoVision XT software. Within the software, virtual vertical lines divided the tank into three equal sections and allowed us to measure the time spent in each section. Both entry and time spent in the left-most segment indicated a preference for proximity to the “opponent,” whereas entry and time spent to the rightmost segments implied avoidance.

### Social Interaction

We analyzed social interaction using a previously described protocol (Gerlai et al., 2000; Gerlai, 2003; Meshalkina et al., 2018). The apparatus comprised three aquaria of the same size (30 cm long × 15 cm high × 10 cm wide) lined up continuously by their 10 cm wall. Hence, there was a “central aquarium,” in which one animal was placed; the “stimulus aquarium,” in which 15 fish were placed as a stimulus; and the “empty aquarium,” which only contained water during the test. The relative position to the center aquarium (i.e., left or right side) in which the stimulus aquarium and the empty aquarium were positioned was counterbalanced. Individual fish were placed in the central aquarium for a total of 6 min. During the first 60 s, we placed an opaque division between the central aquarium and the other aquariums. We did not analyze the habituation period. After this period, we removed the divisions and started the test. We recorded behavior for 5 min and evaluated it using EthoVision XT^®^ tracking software. The software virtually divided the central aquarium into two equal parts. We used the time spent in the area closest to the stimulus aquarium as a measurement of stimulus preference, while we considered time spent in the area closest to the empty aquarium as a measurement of stimulus avoidance.

### Aversive Memory

We evaluated inhibitory avoidance using a glass tank (18 cm long × 9 cm wide × 7 cm high) with two equal-size compartments, designated hereon as dark and white and divided by a sliding guillotine-type partition (9 cm × 7 cm; Blank et al., 2009; Altenhofen et al., 2017b; Bridi et al., 2017). We defined compartments with opaque plastic self-adhesive films in black or white colors that externally covering the walls, the floor, and the corresponding sides of the partition. Two electrodes extending up the wall and placed on each far side of the opposing walls of the dark compartment were attached to an 8 V stimulator that administered a final 3 ± 0.2 V AC shock (intensity measured between electrodes and the center of the dark compartment) when manually activated. Zebrafish were trained and tested individually in the inhibitory avoidance apparatus. We gently placed animals on the white side of the task tank while the partition between compartments was closed. After 1 min of habituation with the new environment, we raised the partition to allow fish to cross to the dark side of the tank through a 1 cm high opening.

For the training session, when animals entered the dark side with their entire body, we closed the sliding partition and administered a pulsed electric shock for 5 s. We then removed fish from the apparatus and placed them in a temporary housing tank. Animals were tested 24 h after training. The test session repeated the training protocol, except that we did not apply the shock, and immediately after animals crossed to the dark compartment, we removed them from the apparatus. During testing, the maximum time animals could spend in the white compartment was 180 s (ceiling time). If that happened, we gently removed animals and recorded their result as 180 s. The latency to completely enter the dark compartment was measured in both sessions. Test latencies were used as an index of memory retention.

### Whole-Body Cortisol Determination

Immediately after treatment, we euthanized animals by hypothermic shock, weighed each fish, and macerated it. Zebrafish trunks were minced and placed into a test tube with 2 ml phosphate-buffered saline (PBS), pH 7.4. We transferred the content to another test tube and added ethyl ether. The tube was vortexed, frozen in liquid nitrogen and the unfrozen portion (ethyl ether containing cortisol) was decanted. The ethyl ether was transferred to a new tube and completely evaporated, yielding a lipid extract containing cortisol. We measured whole-body cortisol in duplicate samples of extracted tissue and determined the concentration by enzyme-linked immunosorbent assay kit (ELISA; EIAgen CORTISOL test, Bio Chem Immuno Systems) from tissue extracts resuspended in PBS (Sink et al., 2007; Idalencio et al., 2015; Oliveira et al., 2017).

### RNA Isolation and Real-Time Quantitative Polymerase Chain Reaction (RT-qPCR)

We euthanized zebrafish by hypothermal shock; we removed brains and evaluated glucocorticoid receptor (*gr*) and corticotropin-releasing factor (*crf*) gene expression using RT-qPCR. Total RNA was isolated from zebrafish brain with TRIzol^®^ Reagent (Life Technologies) following the manufacturer’s instructions. RNA integrity was assessed by visual inspection on a standard 1% agarose gel. After treatment with deoxyribonuclease I (Sigma-Aldrich) to eliminate genomic DNA contamination (following the manufacturer’s instructions), we measured RNA purity (260 nm/280 nm absorbance ratio ~2.0) and concentration with a Nanodrop^®^. We synthesized complementary DNA (cDNA) using the ImProm-II™ Reverse Transcription System (Promega) from 1 μg of total RNA, following the manufacturer’s instructions. We performed RT-qPCR using SYBR^®^ green I (Invitrogen) on a 7500 Real-time PCR System (Applied Biosystems). The PCR cycling conditions were: an initial polymerase activation step for 5 min at 95°C, followed by 40 cycles of 15 s at 95°C for denaturation, 35 s at 60°C for annealing, and 15 s at 72°C for elongation. At the end of the cycling protocol, a melting-curve analysis was included, and fluorescence was measured from 60 to 99°C to confirm the specificity of primers and the absence of primer-dimers. In all cases, there was a single peak. All real-time assays were carried out in quadruplicate and, in all cases, a reverse transcriptase negative control was included to replace templates for DNAse/RNAse-free distilled water in each PCR. *actb1*, *ef1α*, and *rpl13α* served as reference genes for normalization. The primer sequences are: *crf* forward 5′-CAA TTA CGC ACA GAT TCT CCT CG-3′ and reverse 5′-GAA GTA CTC CTC CCC CAA GC-5′ (Khezri et al., 2017); *gr* forward 5′-ACT CCA TGC ACG ACT TGG TG-3′ and reverse 5′-GCA TTT CGG GAA ACT CCA CG-3′ (Manuel et al., 2014); *actb1* forward 5′-CGA GCT GTC TTC CCA TCC A-3′ and reverse 5′-TCA CCA ACG TAG CTG TCT TTC TG-3′ (Tang et al., 2007); *ef1a* forward 5′-CTG GAG GCC AGC TCA AAC AT-3′ and reverse 5′-ATC AAG AAG AGT AGT ACC GCT AGC ATT AC-3′ (Tang et al., 2007); and *rpl131* forward 5′-TCT GGA GGA CTG TAA GAG GTA TGC-3′ and reverse 5′-AGA CGC ACA ATC TTG AGA GCA G-3′ (Tang et al., 2007). We calculated the efficiency per sample using LinRegPCR 2017.0 software^1^. We analyzed the stability and the optimal number of reference genes according to the pairwise variation (*V*) by GeNorm 3.5 Software^2^. We determined relative messenger RNA (mRNA) expression using the 2^−ΔΔCq^ method (Bustin et al., 2013).

### Statistical Analysis

Data are expressed as mean ± standard error of the mean (SEM). For all comparisons, we defined the level of significance as *p* < 0.05. The sample size is in agreement with previously published studies using zebrafish as a model animal (Cachat et al., 2010; Sison and Gerlai, 2011; Ponzoni et al., 2016; Nabinger et al., 2018; Rosa et al., 2018). Novel tank test results were analyzed by one-way analysis of variance (ANOVA), followed by Tukey’s *post hoc* test. Inhibitory avoidance uses a cut-off at 180 s (ceiling time) and we used nonparametric tests. Training and test latencies within each group were compared by the Wilcoxon matched-pairs test. Latencies of multiple groups were compared using Kruskal–Wallis and comparisons between training and test sessions were done with Mann–Whitney *U* tests. For analyses of *crf* and *gr* gene expression, we used ANOVA and Dunn’s multiple-comparison test. We employed GraphPad Prism 8 (La Jolla, CA, USA) software for statistical analyses.

## Results

We used LC-MS/MS to quantify pyriproxyfen in the treated water and detected 0.125, 0.675, and 1.75 mg/l treatments, respectively (Table 1).

**Table 1:** Pyriproxyfen quantification in water using liquid chromatography coupled to tandem mass spectrometry (LC-MS/MS).

Compound	Concentration (mg/L)	Standard deviation
Pyriproxyfen (CAS-95737-68-1)	0.125	0.0070
	0.675	0.0070
	1.75	0.0565

*The data are expressed as mean ± standard deviation (SD; *n* = 3)*.

**Figure 1:**
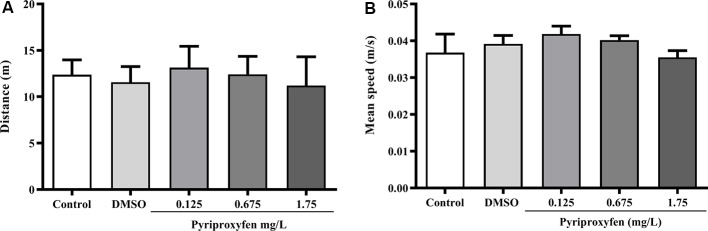
Effects of 96-h pyriproxyfen exposure on locomotor parameters: **(A)** distance and **(B)** mean speed (*n* = 15–21). Data are expressed as the mean ± standard error of the mean (SEM). We analyzed data with a one-way analysis of variance (ANOVA), followed by Tukey’s *post hoc* test.

**Figure 2:**
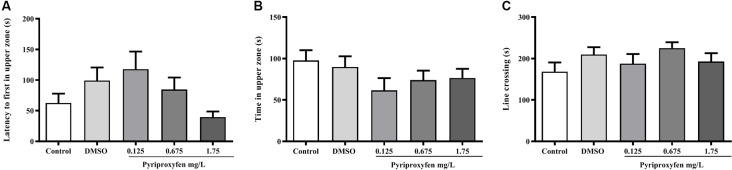
Effects of 96-h pyriproxyfen exposure on **(A)** latency to the upper zone, **(B)** time in the upper zone, **(C)** and line crossings in adult zebrafish (*n* = 16–20). Data are expressed as the mean ± SEM. We analyzed the data with a one-way ANOVA, followed by Tukey’s *post hoc* test.

Pyriproxyfen at all concentrations (0.125, 0.675, or 1.75 mg/l) did not alter locomotor and anxiety parameters after 96 h exposure when compared to control or DMSO groups. There were no significant changes in distance (*F*_(4,83)_ = 1.902, *p* = 0.1178; Figure 1A), mean speed (*F*_(4,83)_ = 0.9983, *p* = 0.4133; Figure 1B), latency to first enter the upper zone (*F*_(4,83)_ = 2.538, *p* > 0.05; Figure 2A), time spent in the upper zone (*F*_(4,83)_ = 0.9834, *p* = 0.4212; Figure 2B), or line crossings (*F*_(4,78)_ = 1.264, *p* = 0.2910; Figure 2C).

**Figure 3:**
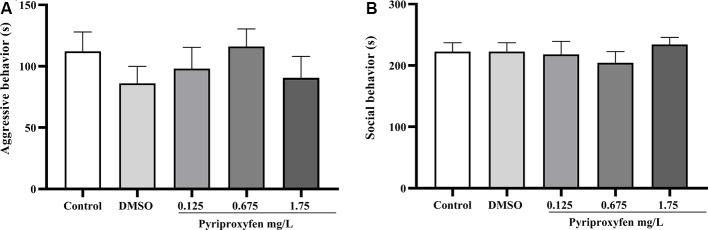
Effects of 96-h pyriproxyfen exposure on **(A)** aggression (*n* = 14–15); and **(B)** social interaction parameters (*n* = 13–15). Data are expressed as the mean ± SEM. We analyzed the data with a one-way ANOVA, followed by Tukey’s *post hoc* test.

Concerning social behavior, pyriproxyfen at 0.125, 0.675, or 1.75 mg/l did not alter aggressive behavior (*F*_(4,69)_ = 0.6852, *p* = 0.6046; Figure 3A) or social interaction (*F*_(4,68)_ = 0.4489, *p* = 0.7728; Figure 3B) after 96 h exposure when compared to control or DMSO groups.

**Figure 4:**
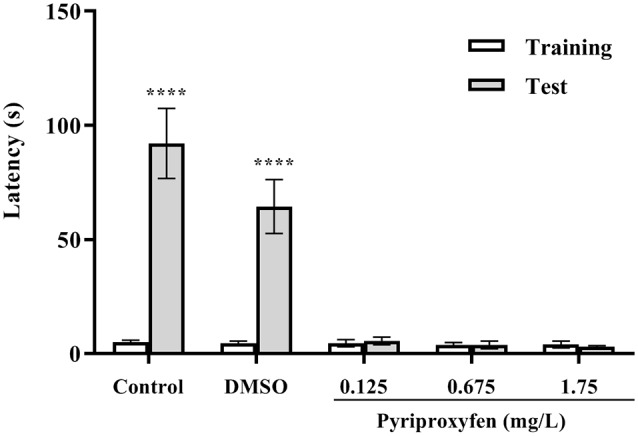
Inhibitory avoidance task performance on the training and long-term memory test session 96 h after pyriproxyfen exposure (*n* = 15). Data are presented as mean ± SEM.We found no differences between training performance among all exposed groups as evaluated by the Kruskal–Wallis test. The asterisk indicates the difference between training and test sessions for each group, compared using the Wilcoxon matched-pair test (****p < 0.0001).

The inhibitory avoidance test showed that animals in the control and DMSO groups presented an increase in latency to move to the dark side of the aquarium (*U* = 0, *p* < 0.0001 and *U* = 0, *p* < 0.0001, respectively). However, we observed aversive memory deficits in fish exposed to pyriproxyfen at concentrations of 0.125 mg/l (*U* = 102.5, *p* = 0.6852), 0.675 mg/l (*U* = 83.00, *p* = 0.2053), and 1.75 mg/l (*U* = 100.5, *p* = 0.6210). There were no differences in the latencies in the training and test sessions (Figure 4). Moreover, there were no differences between training and test latencies. Hence, these data demonstrated that memory retention in fish exposed to pyriproxyfen was impaired.

Inhibitory avoidance task performance on the training and long-term memory test session 96 h after pyriproxyfen exposure (n = 15). Data are presented as mean ± standard error of the mean (SEM). We found no differences between training performance among all exposed groups as evaluated by the Kruskal-Wallis test. The asterisks indicate the difference between training and test sessions for each group, compared using the Wilcoxon matched-pair test (****p < 0.0001).

**Figure 5:**
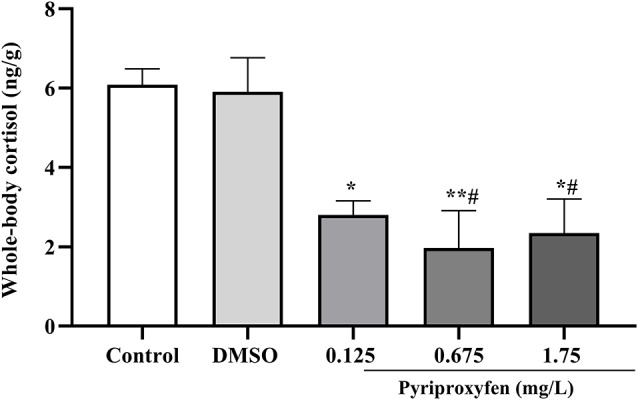
Whole-body cortisol response of zebrafish exposed to pyriproxyfen for 96 h (*n* = 3–6). Data are expressed as mean ± SEM and analyzed by one-way ANOVA, followed by Tukey’s multiple comparisons test.The symbols *p < 0.05 and **p < 0.005 indicate differences in relation to the control whereas ^#^*p* < 0.05 presents difference when compared to the vehicle group.

There was a decrease of cortisol levels at all tested pyriproxyfen concentrations (0.125, 0.675, and 1.75 mg/l) when compared to the control group. By contrast, cortisol was only reduced in relation to vehicle at 0.675 and 1.75 mg/l pyriproxyfen concentrations (*F*_(4,17)_ = 8.737, *p* = 0.0005; Figure 5).

**Figure 6:**
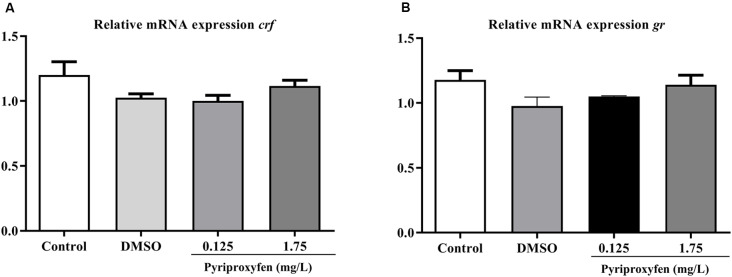
Effects of 96-h pyriproxyfen exposure on **(A)** *crf* and **(B)** *gr* gene expression (*n* = 9–12). Data are expressed as mean ± SEM and analyzed by one-way ANOVA, followed by Dunn’s *post hoc* test (*p* > 0.05).

We evaluated the effects of 0.125 and 1.75 mg/l pyriproxyfen exposure on *crf* (*F*_(3,41)_ = 2.366, *p* = 0.0849; Figure 6A) and *gr* (*F*_(3,41)_ = 1.982, *p* = 0.1317; Figure 6B) gene expression. Pyriproxyfen did not significantly alter *crf* or *gr* mRNA transcript levels compared to the control or DMSO groups.

## Discussion

In this study, we found that adult zebrafish exposed to pyriproxyfen, a widely used insecticide, showed reduced levels of cortisol and impaired aversive memory. First, we quantified pyriproxyfen in the water through LC-MS/MS to certify that the animals were exposed to the compound throughout the entire period, and thus the effects would result from the96-h exposure.

Studies conducted to evaluate the action of different toxic agents—for example, tebuconazole, dichlorvos, and glyphosate—have shown that the locomotor behavior of exposed adult zebrafish is significantly altered (Altenhofen et al., 2017a, 2019; Bridi et al., 2017). In contrast to those studies, we found that locomotor parameters, such as distance and mean speed, were not altered by pyriproxyfen exposure. Moreover, the common variations associated with anxiety, such as time spent in and latency to enter the upper zone, as well as entries in the upper zone (measured through line crossing), were not altered by pyriproxyfen exposure. These data indicate that the cognitive deficits presented by animals exposed to pyriproxyfen are not related to changes in locomotor or anxiety parameters. Moreover, the deficits observed cannot be attributed to the diluent (DMSO) because there was no difference between the control and DMSO groups in the experiments. Several studies have demonstrated that DMSO is characterized as a safe diluent, with no toxic effects in zebrafish, when used in low concentrations (Hallare et al., 2006; Nery et al., 2014). Also, zebrafish exposed to pyriproxyfen showed no changes in social behaviors, such as aggression and social interaction, both of which are used to analyze disorders of the central nervous system in translational studies (Gerlai, 2014).

To the best of our knowledge, this study is the first to report the effects of pyriproxyfen on memory parameters and cortisol levels in adult zebrafish. The inhibitory avoidance task evaluates aversive memory; during the training session, the animals should learn that crossing to the dark compartment is associated with a shock. This context is related to the fact that a zebrafish prefers dark environments in its adult life; this behavior aims to protect it from possible predators. So, when the animal is placed in the white compartment, it is expected that when opening the sliding guillotine-type partition it will move quickly to the dark compartment, for its own protection. During the training session, the animals receive a shock; hence, it is expected that 24 h later, throughout the test, this latency of passage to the dark side will increase. If the animal presents some memory deficit, this latency is not increased, and these data show that the animal is unable to retain the memory related to the shock (Blank et al., 2009). Our data showed that pyriproxyfen induced a deficit in the retention of aversive memory at all tested doses.

The learning experience of an aversive memory (as the inhibitory avoidance task in which we observed memory impairment) initiates the release of stress hormones such as cortisol. This increase in cortisol levels is central in modulating memory consolidation (for a review see McGaugh, 2000) as this hormone can act throughout the brain by binding to glucocorticoid receptors and improving memory performance in rodents and humans (Goosens, 2012). In humans, administration of 11-beta-hydroxylase (an inhibitor of cortisol synthesis) presented cortisol reduction and memory impairment (Rimmele et al., 2015). Manuel et al. (2014) showed that zebrafish with the highest levels of cortisol performed better in memory tests 24 h after training. In our experiments, zebrafish exposed to all concentrations of pyriproxyfen showed both aversive memory impairment and lower cortisol levels. Whole-body cortisol levels were measured in different animals from those submitted to the inhibitory avoidance task. Therefore, correlating memory performance and cortisol levels was not possible and this is one limitation of this study. However, cortisol levels and the training session of inhibitory avoidance task were measured in animals submitted to the same conditions and immediately after pyriproxyfen treatment. Therefore, it is expected that cortisol levels are similar in animals submitted for both analyses. Due to the pivotal effect of cortisol in memory consolidation, lower cortisol levels may be impairing the performance in the task. Although it is already well established that cortisol influences memory, it is not possible to assume there is a direct causal relationship between our findings in aversive memory impairment and lower cortisol levels. Further studies are required to investigate the mechanisms involved in the memory deficit induced by pyriproxyfen, especially its influence on the cholinergic system since acetylcholine has a pivotal role in memory and behavior.

Cortisol is important for maintaining homeostasis and an imbalance in the HPI axis can trigger changes in several physiological parameters (Späth-Schwalbe et al., 1991; Bennion et al., 2015; De Quervain et al., 2017). In the context of aversive stimuli, the neurons of the PVH (or the NPO in fish) initiate CRF secretion, which leads to the secretion of glucocorticoid (e.g., cortisol) and its subsequent effect on the HPI axis (Goosens, 2012). Although, we demonstrated that cortisol was significantly reduced in animals exposed to pyriproxyfen, we did not observe changes in the expression of *gr* and *crf*. Consistent with our data, a study showed that zebrafish submitted to stressful tasks for 7 and 14 days, with memory deficits and changes in cortisol levels, did not demonstrate changes in *crf* expression (Manuel et al., 2014). It is important to note that for the short- or long-term memory process to function properly, it is crucial that cortisol easily access the brain and bind to mineralocorticoid (MR) and glucocorticoid (GR) receptors. However, low cortisol levels may not be related to decreased expression of these receptors, as previous studies and our data indicate.

In summary, our study demonstrated that pyriproxyfen exposure impaired inhibitory avoidance memory and altered cortisol levels in zebrafish. Such changes may significantly affect the survival of fish in natural habitats, which may, in turn, unbalance the whole ecosystem and affect a broad spectrum of aquatic organisms. Further research with this compound is needed to characterize the interaction sites between pyriproxyfen and the endocrine and central nervous systems. The present study underlines that zebrafish have the potential to be used in translational studies linked with learning and memory in animals submitted to chemical compounds exposure.

## Data Availability Statement

The original contributions presented in the study are included in the article, further inquiries can be directed to the corresponding author.

## Ethics Statement

The animal studies were reviewed and approved by the Institutional Animal Care Committee from Pontificia Universidade Católica do Rio Grande do Sul (CEUA-PUCRS, protocol number 7546/2016).

## Author Contributions

DG and CB contributed to the study design. DG, SA, JG, and GR performed behavioral and cortisol assays. LK and MB performed gene expression analysis. DG and CB wrote the first draft of the manuscript. All authors contributed to manuscript revision and read and approved the submitted version.

## Conflict of Interest

The authors declare that the research was conducted in the absence of any commercial or financial relationships that could be construed as a potential conflict of interest.

## Footnotes

^1^https://LinRegPCR.nl

^2^https://genorm.cmgg.be/
